# Greater Breadth of Vaccine-Induced Immunity in Females than Males Is Mediated by Increased Antibody Diversity in Germinal Center B Cells

**DOI:** 10.1128/mbio.01839-22

**Published:** 2022-07-20

**Authors:** Rebecca L. Ursin, Santosh Dhakal, Hsuan Liu, Sahana Jayaraman, Han-Sol Park, Harrison R. Powell, Morgan L. Sherer, Kirsten E. Littlefield, Ashley L. Fink, Zexu Ma, Alice L. Mueller, Allison P. Chen, Kumba Seddu, Yishak A. Woldetsadik, Patricia J. Gearhart, H. Benjamin Larman, Robert W. Maul, Andrew Pekosz, Sabra L. Klein

**Affiliations:** a Department of Biochemistry and Molecular Biology, Johns Hopkins Bloomberg School of Public Health, Baltimore, Maryland, USA; b W. Harry Feinstone Department of Molecular Microbiology and Immunology, Johns Hopkins Bloomberg School of Public Health, Baltimore, Maryland, USA; c Department of Pathology, Johns Hopkins School of Medicine, Baltimore, Maryland, USA; d Laboratory of Molecular Biology and Immunology, National Institute on Aging, National Institutes of Health, Baltimore, Maryland, USA; Washington University School of Medicine

**Keywords:** sex difference, antibody response, H1N1, viral immunity, antigenic drift, antibody function, antigenic variation

## Abstract

Inactivated influenza vaccines induce greater antibody responses in females than males among both humans and mice. To test the breadth of protection, we used recombinant mouse-adapted A/California/2009 (maA/Cal/09) H1N1 viruses containing mutations at one (1M), two (2M), or three (3M) antigenic sites, in addition to a virus containing the 1M mutation and a substitution of the Ca2 antigenic site (Sub) with one derived from an H5 hemagglutinin (HA) to challenge mice of both sexes. Following maA/Cal/09 vaccination, females produced greater virus-specific, class-switched total IgG and IgG2c antibodies against the vaccine and all mutant viruses, and antibodies from females recognized a greater number of unique, linear HA epitopes than did antibodies from males. While females had greater neutralizing antibody titers against the vaccine virus, both sexes showed a lower neutralization capacity against mutant viruses. After virus challenge, vaccinated females had lower pulmonary virus titers and reduced morbidity than males for the 1M and 2M viruses, but not the Sub virus. Females generated greater numbers of germinal center (GC) B cells containing superior somatic hypermutation (SHM) frequencies than vaccinated males. Deletion of activation-induced cytidine deaminase (*Aicda*) eliminated female-biased immunity and protection against the 2M virus. Harnessing methods to improve GC B cell responses and frequencies of SHM, especially in males, should be considered in the development of universal influenza vaccines.

## INTRODUCTION

Infection with influenza A virus (IAV) or influenza B virus (IBV) is seasonal, and the pathogenesis, antigenicity, and virulence of these viruses change seasonally. Influenza viruses have segmented RNA genomes, and this enables the evolution of antigenically distinct viruses that can evade immunological memory (1–3). The propensity for influenza viruses to undergo antigenic drift results in the need for annual updates to the seasonal influenza vaccine virus strain selection. Annual vaccination prevents severe disease and the spread of the virus; the available vaccination platforms, however, can vary widely in their effectiveness and protection (4, 5). Most of the worldwide population receives an inactivated influenza vaccine (IIV) that targets the influenza virus attachment protein, hemagglutinin (HA). IIVs include either three strains (trivalent; one H1N1, one H3N2, and one IBV) or four strains (quadrivalent; the same as trivalent but with two IBV lineages), but each strain can independently vary in effectiveness (6, 7). For example, during the 2017 to 2018 influenza season, a mismatch in the H3N2 circulating and selected vaccine strains was associated with egg adaptation mutations of the vaccine virus, leading to an influenza season as severe as the 2009 H1N1 pandemic season in the United States (8).

Improved influenza vaccine effectiveness requires a better understanding of both viral and host factors that affect vaccine-induced immune responses (9–11). Viral factors include egg adaptation-associated changes to the vaccine virus, which can arise during vaccine manufacturing; seasonal antigenic drift stemming from immunological pressure from the host; genetic shift of influenza viruses; and posttranslational modifications of the virus (e.g., addition or loss of glycosylation sites) (6, 12). There are biological host variables that also influence vaccine effectiveness and protection; these include but are not limited to biological sex, age, reproductive status, and body mass index (13, 14). In particular, biological sex is a predictor of antibody responses and protection following receipt of influenza vaccines, with females generally producing greater antibody responses than males. These observed sex differences are age dependent and associated with circulating sex steroid hormone concentrations (15, 16).

For many vaccines, including IIV, the primary correlate of protection is the antibody response generated by B cells (5). After immunization with IIV, female mice exhibit a greater quantity and quality of vaccine-specific antibodies and protection from both infection and severe disease than males (8, 17–20). While being female is a predictor of greater immunity and protection against influenza virus infection following vaccination, few studies have considered sex differences in protection against influenza in the context of evolved virus mutations, which we have shown is a limitation to female-biased protection against influenza virus in humans (8). Antibody maturation selects for specificity over cross-reactivity of B cell-mediated immunity (i.e., affinity maturation), which may limit the breadth of female-biased immunity when faced with rapid mutations in RNA viruses such as influenza virus. Most antibodies directed toward influenza viruses recognize the major antigenic sites on the immunodominant HA protein, which for H1 viruses include the Sa, Sb, Ca1, Ca2, and Cb sites (21–23). Antisera from A/Michigan/45/2015-infected mice, for example, contain antibodies that recognize sites Sb and Ca2 to a greater degree than the other sites (24).

Using viruses with mutations in some of these major antigenic sites of the HA protein of mouse-adapted A/California/04/2009 H1N1 (maA/Cal/09) (19), we tested the hypothesis that female-biased immunity and protection would be dependent on the extent of virus diversity as well as class switch recombination (CSR) and somatic hypermutation (SHM) mechanisms in B cells, which constrains the breadth of epitope recognition. Neutralization of mutant maA/Cal/09 viruses was equally poor in vaccinated male and female mice, despite vaccinated females being better protected against these viruses. Vaccinated females benefited from the greater production of class-switched, somatically hypermutated antibodies generated in germinal center (GC) B cells, which allowed females to recognize more diverse maA/Cal/09 HA antigens epitopes to a greater degree than vaccinated males. The female-biased protection against influenza virus infection and disease after vaccination was driven by differential mechanisms in males versus females. There were, however, limits to the female-biased immunity and protection that were dependent on the extent of virus mutations.

## RESULTS

### HA mutations in maA/Cal/09 viruses reduce virus growth *in vitro* and cause greater morbidity in naive females than males *in vivo*.

**FIG 1:**
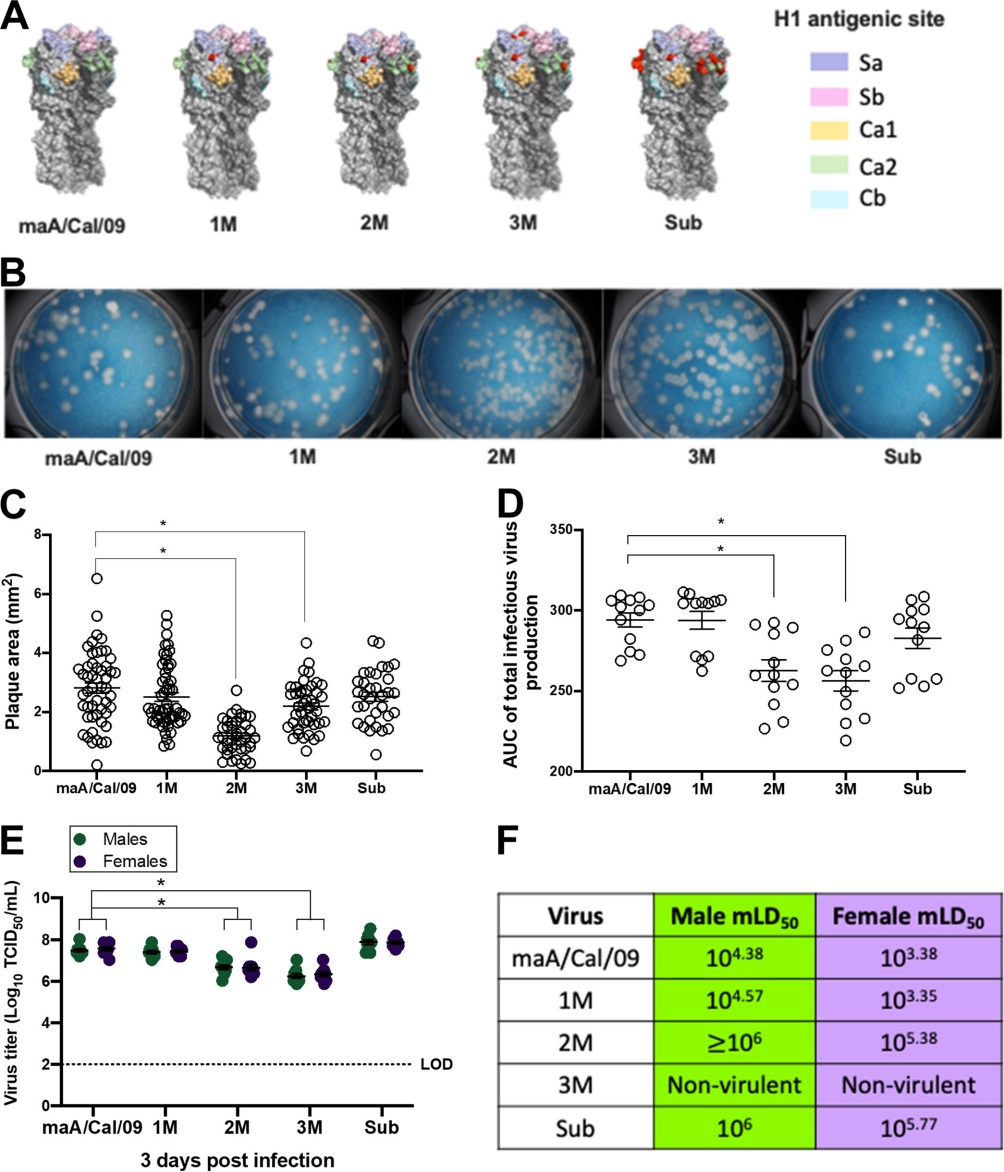
Mutations in mouse-adapted A/California/4/2009 H1N1 viruses cause differences in growth morphology and kinetics *in vitro* and sex differences in virulence *in vivo*. (A) Mutations were inserted into antigenic sites of the parental mouse-adapted A/California/4/2009 (maA/Cal/09) H1N1 virus hemagglutinin (HA). Single point mutations were inserted into the Sa site (single mutant, 1M); Sa and Ca2 sites (double mutant, 2M); and Sa, Ca2, and Sb sites (triple mutant, 3M). A fourth virus had a single point mutation in the Sa site at the same position as 1M but had a nonhuman, avian H5 sequence substituted into the Ca2 region (Sub). The H1 major antigenic sites are shown in pastel shades on the head of the HA protein trimers (different shades of gray). Mutations are shown in red. The HA structures were created in PyMOL with PDB 3LZG for A/California/4/2009. (B) Viruses were used to perform plaque assays to determine growth and plaque morphology on a monolayer of MDCK cells. (C) Plaque sizes for each virus were measured and are plotted as area per square millimeter. (D) Low-MOI growth curves were used to compare the growth kinetics of the parental maA/Cal/09 virus and each of the mutant viruses, with the AUC for virus growth calculated up to 48 h postinfection (i.e., peak titers). (E) Lungs were collected from naive (unvaccinated) male and female C57BL/6CR mice 3 days after infection with 10^5^ TCID_50_ units of each mutant virus and used to measure replicating virus (*n* = 9 to 10/sex/virus). (F) Log_10_ dilutions of each virus ranging from 10 to 10^6^ TCID_50_ were used to intranasally infect male and female mice (*n* = 5 to 10/sex/virus dose) to calculate the mLD_50_. Bars represent the averages and standard errors of the means (SEM); LOD is the limit of detection for the assay. *, *P < *0.05.

To test the breadth of female-biased antibody-mediated immunity against antigenically diverse viruses, naturally occurring mutations observed in human A/Cal/09 (23, 24) antigenic sites were introduced into the HA sequence of maA/Cal/09: (i) one point mutation (1M) in Sa; (ii) two point mutations (2M) in Ca2 and Sa; (iii) three point mutations (3M) in Ca2, Sa, and Sb; and (iv) a point mutation in Sa along with an antigenic site substitution of the Ca2 epitope with one derived from an H5 sequence (Sub) (Fig. 1A; see also Table S1 in the supplemental material). Overall plaque morphology of the parental maA/Cal/09 virus included medium-sized plaques with complete clearance (i.e., cell death) within the middle of the plaque. While the 1M and Sub viruses had a plaque morphology similar to that of maA/Cal/09, the 2M and 3M virus plaques were significantly smaller (Fig. 1B and C). Low-multiplicity of infection (MOI) growth curves showed that the parental maA/Cal/09 virus grew better by 48 h postinfection (peak titer) than the 2M and 3M viruses (Fig. 1D). The mutant maA/Cal/09 viruses were each able to replicate and be released by Madin-Darby canine kidney (MDCK) cells, albeit to different peak titers and with different plaque morphologies.

10.1128/mbio.01839-22.4 TABLE S1 Amino acid mutations in the HA of maA/Cal/09 that were inserted to create the 1M, 2M, 3M, and Sub viruses for these studies. Download Table S1, DOCX file, 0.1 MB. Copyright © 2022 Ursin et al. 2022 Ursin et al. https://creativecommons.org/licenses/by/4.0/ This content is distributed under the terms of the Creative Commons Attribution 4.0 International license.

To assess the virulence of the mutant viruses, naive male and female mice were intranasally infected with increasing doses (10^1^ to 10^6^ 50% tissue culture infective dose [TCID_50_]) of either the parental maA/Cal/09 virus or one of the four mutant viruses and either euthanized 3 days postinfection to measure pulmonary virus titers (from mice infected with 10^5^ TCID_50_ only) or followed for 21 days to quantify morbidity (i.e., body mass loss) and mortality. Following infection with 10^5^ TCID_50_ of each virus, naive males and females exhibited similar pulmonary titers to all infecting viruses (Fig. 1E). The parental maA/Cal/09 and 1M and Sub viruses replicated to higher titers in the pulmonary tissue than the 2M or 3M viruses (Fig. 1E). Dose-dependent survival curves were used to calculate the mouse 50% lethal dose (mLD_50_) for each virus separately for males and females (Fig. 1F). For the maA/Cal/09, 1M, and 2M viruses and to a lesser extent Sub virus, naive female mice showed greater mortality than male mice, with females having an mLD_50_ that was approximately 1 log lower than that for males (Fig. 1F). The 3M virus did not cause mortality at any dose in either sex of mice and was labeled as nonvirulent. As these viruses acquired more mutations, becoming antigenically more distinct from the parental virus, virulence and sex differences in mortality were reduced among naive mice.

### Vaccinated females have greater antibody cross-reactivity against mutant maA/Cal/09 viruses than males.

**FIG 2:**
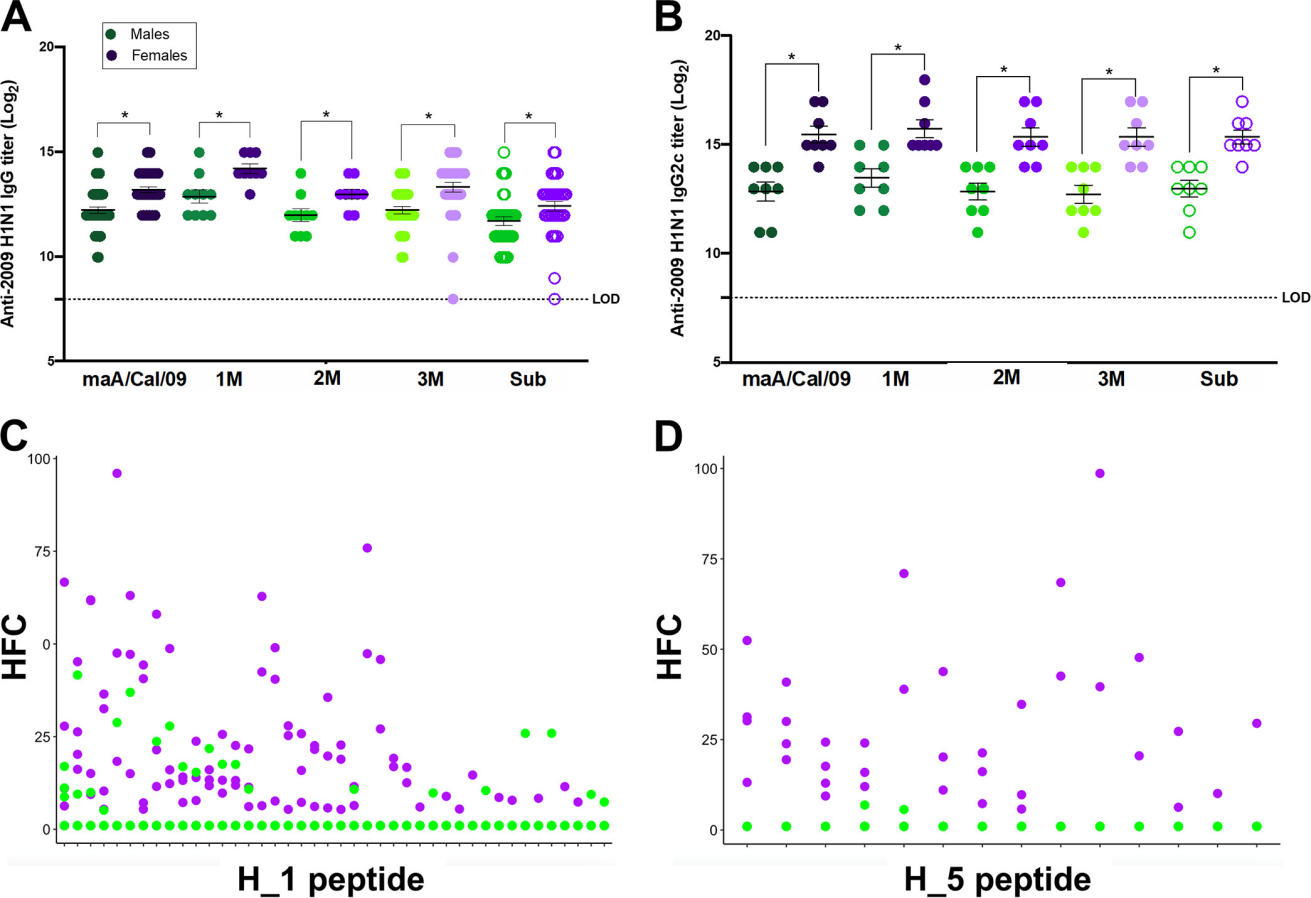
Vaccinated female mice mount greater cross-reactive antibody responses to mutant mouse-adapted A/California/4/2009 H1N1 viruses than males. Male and female C57BL/6CR mice were vaccinated and boosted with mouse-adapted A/California/4/2009 (maA/Cal/09) H1N1 virus and bled at 28 days postvaccination. (A and B) Serum was used to measure cross-reactive IgG (*n* = 9 to 45/sex) (A) and IgG2c (*n* = 8/sex) (B) antibodies toward the vaccine virus maA/Cal/09 and the mutant viruses 1M, 2M, 3M, and Sub by enzyme-linked immunosorbent assay (ELISA). Serum from vaccinated males and females was used to determine antibody reactivity to thousands of linear influenza hemagglutinin epitopes via PhIP-Seq using the VirScan library. (C and D) Reactivity of these vaccine-induced antibodies to unique H1 epitopes (C) and cross-reactivity to H5 epitopes (D) are shown (*n* = 9 to 10/sex), with each unique epitope represented on the *x* axis and the hits fold change (HFC) on the *y* axis. HFC is a measure of bound peptide from the serum-immunoprecipitated samples relative to the mock-immunoprecipitated samples, with no significant difference set to an HFC of 1 (see Text S1 in the supplemental material). Bars represent the averages and SEM; LOD is the limit of detection for the assay. *, *P < *0.05.

Previous studies have shown that female C57BL/6 mice produce greater anti-mA/Cal/09 total IgG and IgG2c, but not IgG1, titers than male mice following vaccination (15, 19). In C57BL/6 mice, IgG2c is the isotype that is produced in highest titers and affords the greatest level of protection against infection with IAVs (19). To determine if sex differences were observed in antibody recognition of mutant viruses, mice were vaccinated and boosted with maA/Cal/09, and serum was used to measure cross-reactive total IgG and IgG2c antibodies against maA/Cal/09, 1M, 2M, 3M, and Sub viruses. Vaccinated females produced greater anti-maA/Cal/09 total IgG (Fig. 2A) and IgG2c (Fig. 2B) titers than males and had greater cross-reactive total IgG (Fig. 2A) and IgG2c (Fig. 2B) against the 1M, 2M, 3M, and Sub viruses than males.

10.1128/mbio.01839-22.1 TEXT S1 Materials and methods for plaque assays, virus growth curves, vaccine generation, antibody assays, flow cytometry, immunofluorescence, B-cell isolation, PCR and sequencing, and related references. Download Text S1, DOCX file, 0.03 MB. Copyright © 2022 Ursin et al. 2022 Ursin et al. https://creativecommons.org/licenses/by/4.0/ This content is distributed under the terms of the Creative Commons Attribution 4.0 International license.

Greater breadth of recognition of HA viral epitopes might explain female-biased cross-reactivity against mutant maA/Cal/09 viruses. To evaluate recognition of linear viral epitopes, bacteriophage immunoprecipitation sequencing (PhIP-Seq) was performed using the VirScan library, whereby serum from vaccinated male and female mice was combined with a library of linear epitopes from IAV proteins. In total, 75 unique, linear HA epitopes were recognized by the vaccine-induced antibodies produced in at least 1 mouse/sex, with antibodies recognizing 42 H1 epitopes, 4 2009 H1 epitopes, 11 H2 epitopes, 14 H5 epitopes, and 8 H3 epitopes (see Fig. S1A in the supplemental material). Each of 75 epitopes mapped to the HA2 or stem domain, where linear epitopes are most abundant. The binding footprint for the four 2009 H1 epitopes (three of which are unique) is illustrated in Fig. S1B. The percentage of female mice with reactivity to at least one HA epitope was greater than that for males (see Fig. S1A). The hits fold change (HFC) values, which are a measure of bound peptide from the serum-immunoprecipitated samples relative to the mock-immunoprecipitated samples, was greater for females than males for H1 epitopes (Fig. 2C), H5 epitopes (Fig. 2D), and H2 epitopes (see Fig. S1C), which are all group 1 IAVs. Serum from vaccinated females also was significantly better at recognizing H3 epitopes (see Fig. S1D), which is a group 2 IAV, providing evidence of greater potential for heterologous immunity in vaccinated females than males (20). Because females have greater cross-reactivity of antibodies against novel HA epitopes, vaccinated females may have a greater breadth of immunity against IAVs than males.

10.1128/mbio.01839-22.2 FIG S1 Further documenting of the greater reactivity to linear HA stem-based epitopes in antibodies from vaccinated female compared with male mice. Download FIG S1, DOCX file, 0.3 MB. Copyright © 2022 Ursin et al. 2022 Ursin et al. https://creativecommons.org/licenses/by/4.0/ This content is distributed under the terms of the Creative Commons Attribution 4.0 International license.

### Vaccinated females have greater cross-protection against infection and disease than males following challenge with mutant viruses, which is dependent on B cells.

**FIG 3:**
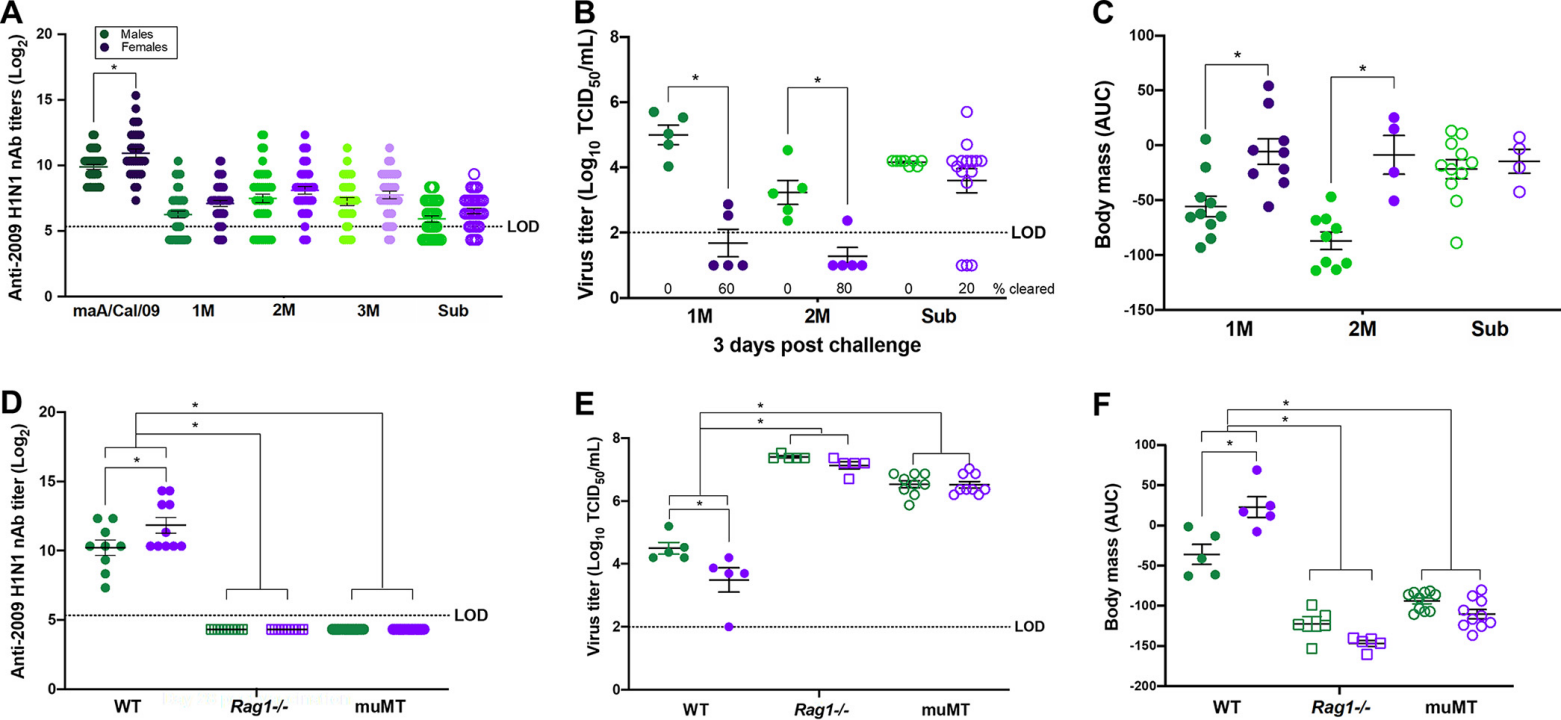
Female-biased, vaccine-induced protection from disease and virus replication following live virus challenge is dependent upon B cells. Male and female mice were vaccinated and boosted with mouse-adapted A/California/4/2009 (maA/Cal/09) H1N1 virus and challenged with 10^5^ TCID_50_ of the virulent mutant viruses 1M, 2M, or Sub. (A) nAb toward each of the five viruses were measured in serum samples collected at 28 days postvaccination from wild-type (WT) C57BL/6CR mice (*n* = 33 to 45/sex). (B) Subset of WT male and female mice were euthanized at 3 days postchallenge to measure replicating virus titers in the lungs (*n* = 5 to 15/sex). (C) Body mass loss after virus challenge was tracked as a correlate for morbidity for 14 days and converted into AUC values, with lower values indicating greater mass loss (*n* = 4 to 11/sex). Male and female WT, *Rag1^−/−^*, and muMT mice on a C57BL/6J background were vaccinated and boosted with maA/Cal/09 H1N1 virus. (D) Serum from 28 days postvaccination was used to measure nAb titers to the maA/Cal/09 H1N1 vaccine virus in knockout mice compared with WT male and female mice (*n* = 10 to 20/sex). Vaccinated and boosted WT, *Rag1^−/−^*, and muMT male and female mice were challenged with 10^5^ TCID_50_ of the 2M mutant virus, and subsets of mice were used to measure replicating virus titers in the lungs at 3 days postchallenge (*n* = 5 to 9/sex). (F) After virus challenge, WT, *Rag1^−/−^*, and muMT male and female mice were monitored for body mass loss for 14 days, with lower AUCs indicating greater mass loss (*n* = 5 to 10/sex). Bars represent the averages and SEM; LOD is the limit of detection for the assay. *, *P < *0.05.

Neutralizing antibodies (nAb), which recognize primarily conformational epitopes (11, 25), were measured as a correlate of protection. Although vaccinated females had greater nAb titers than males against the maA/Cal/09 vaccine virus (19), nAb titers against the mutant maA/Cal/09 viruses were significantly reduced and sex differences were not apparent (Fig. 3A). If live virus neutralization is the correlate of vaccine-induced protection, then females vaccinated against maA/Cal/09 may not be better protected against mutant maA/Cal/09 viruses. To test this hypothesis, vaccinated males and females were challenged with lethal doses of each of the virulent mutant viruses (1M, 2M, and Sub). Subsets of mice were euthanized at 3 days postchallenge to measure peak pulmonary virus titers, while other mice were followed for 14 days for assessment of body mass loss as a measure of morbidity. Following live virus challenge, vaccinated females had lower pulmonary titers of the 1M and 2M viruses, but not the Sub virus, than vaccinated males, with 60% (3/5) and 80% (4/5) of female mice clearing the 1M and 2M virus, respectively, compared with none of the male mice clearing virus (Fig. 3B). Body mass loss over the 14 days postchallenge was translated into an area under the curve (AUC) to show individual data points, with greater mass loss indicated by lower AUC values. Males challenged with either the 1M or 2M virus experienced greater body mass loss (i.e., had lower AUC values) than their female counterparts (Fig. 3C). Challenge with the Sub virus did not cause sex differences in morbidity among vaccinated mice. Despite causing greater overall levels of virus replication in the lungs (Fig. 3B), the Sub virus caused minimal disease in vaccinated males and females (Fig. 3C).

Because live virus neutralization did not predict the female-biased protection against mutant maA/Cal/09 viruses, we sought to ensure that vaccine-induced protection was B cell and not T cell mediated. Male and female wild-type (WT), *mu*MT, and *Rag1^−/−^* mice were vaccinated and boosted with inactivated maA/Cal/09, and antibody responses as well as outcomes following lethal 2M challenge were analyzed. After vaccination, nAb titers against the maA/Cal/09 vaccine virus were significantly greater among WT females than males (Fig. 3D). In the absence of B cells, either alone or in combination with T cells, nAb titers were not detectable in either sex (Fig. 3D). Live 2M virus challenge of vaccinated WT mice resulted in lower pulmonary virus titers and reduced body mass loss among females than males (Fig. 3E and F). In contrast, among both *mu*MT and *Rag1^−/−^* mice, 2M virus titers in the lungs as well as morbidity were greater than among WT mice and sex differences were no longer observed (Fig. 3E and F). Vaccinated *mu*MT and *Rag1^−/−^* mice had similar outcomes following challenge with live 2M virus, suggesting that in the absence of B cells, with or without T cells, vaccine-induced, female-biased protection against virus challenge was eliminated.

### Splenic GC size and frequencies of B cells are greater in vaccinated female than male mice.

**FIG 4:**
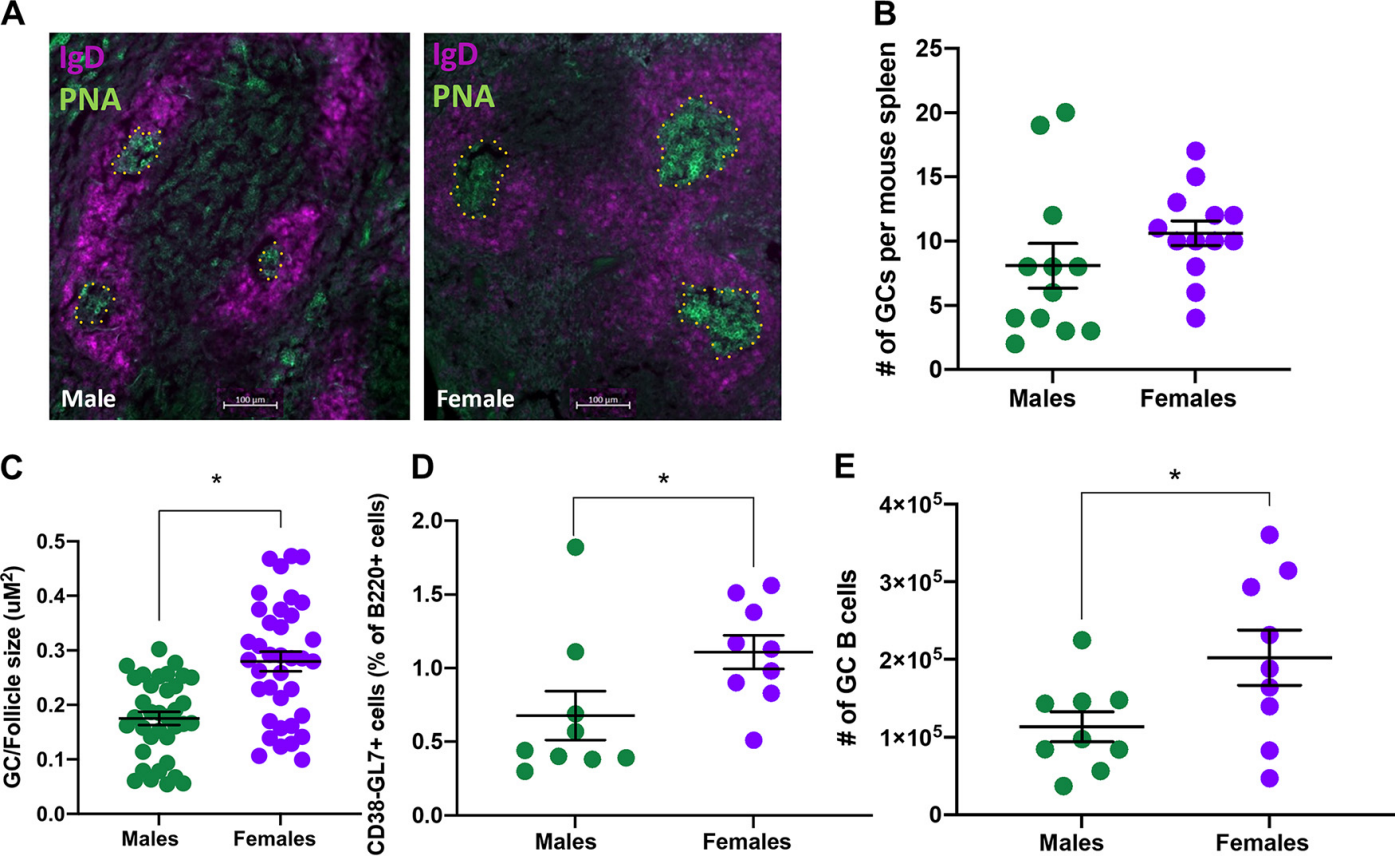
Influenza vaccination elicits a more robust germinal center B cell response in female compared to male mice. Male and female C57BL/6CR mice were vaccinated and boosted with mouse-adapted A/California/4/2009 (maA/Cal/09) H1N1. (A) At 35 days postvaccination, mice were euthanized, and spleens were dissected for gross histology and microscopy at 10× magnification; IgD^+^ cells stained magenta and PNA^+^ cells stained green. GCs are outlined by a yellow stippled line. (B) Total number of GCs per spleen was quantified (*n* = 12 to 13/sex) from 3 sections per animal. (C) Relative sizes of GCs (IgD*^−^*, PNA^+^) relative to its surrounding follicle (IgD^+^, PNA*^−^*) were determined for 36 GCs from males and 38 GCs from females (*n* = 5/sex). (D and E) The percentages of B cells that were B220^+^ were measured via flow cytometry (D), together with the total number of GC B cells in male and female mice after vaccination (*n* = 9/sex) (E). Bars represent the averages and SEM. *, *P < *0.05.

Following vaccination with IIV, antibodies undergo affinity maturation to increase the overall specificity, functionality, and quantity of antibodies produced in GCs in lymph nodes (LNs) and the spleen (26, 27). Because vaccinated female mice produced more total IgG and IgG2c following vaccination (Fig. 2A and B) and experienced greater protection from virus-induced infection and disease (Fig. 3B and C), we hypothesized that females may have greater GC activity than males. Male and female mice were vaccinated and boosted with maA/Cal/09, and whole spleens were dissected at 35 days postvaccination. Spleens from vaccinated male and female mice were frozen, sectioned, and stained to outline total B cell areas (IgD^+^, peanut agglutinin positive [PNA^+^]) and GCs (IgD*^−^*, PNA+) (Fig. 4A). While the overall number of GCs in spleen sections from vaccinated males and females was not different (Fig. 4B), the percentage of the GC area relative to the follicle area was significantly greater in spleens from females than males (Fig. 4C). Both the percent of B220^+^ GC B cells (Fig. 4D; see also Fig. S2) as well as the total number of GC B cells (Fig. 4E; see also Fig. S2) in the spleen were greater in vaccinated females than males. These data suggest that greater activation of GC B cells underlies greater antibody CSR and possibly SHM to result in greater cross-protection against mutated maA/Cal/09 viruses in vaccinated females than males.

10.1128/mbio.01839-22.3 FIG S2 Gating strategy used to identify germinal center B cells from spleens of vaccinated male and female mice. Download FIG S2, DOCX file, 0.3 MB. Copyright © 2022 Ursin et al. 2022 Ursin et al. https://creativecommons.org/licenses/by/4.0/ This content is distributed under the terms of the Creative Commons Attribution 4.0 International license.

### Increased antibody maturation underlies greater vaccine-induced protection against mutant maA/Cal/09 viruses in females than males.

**FIG 5:**
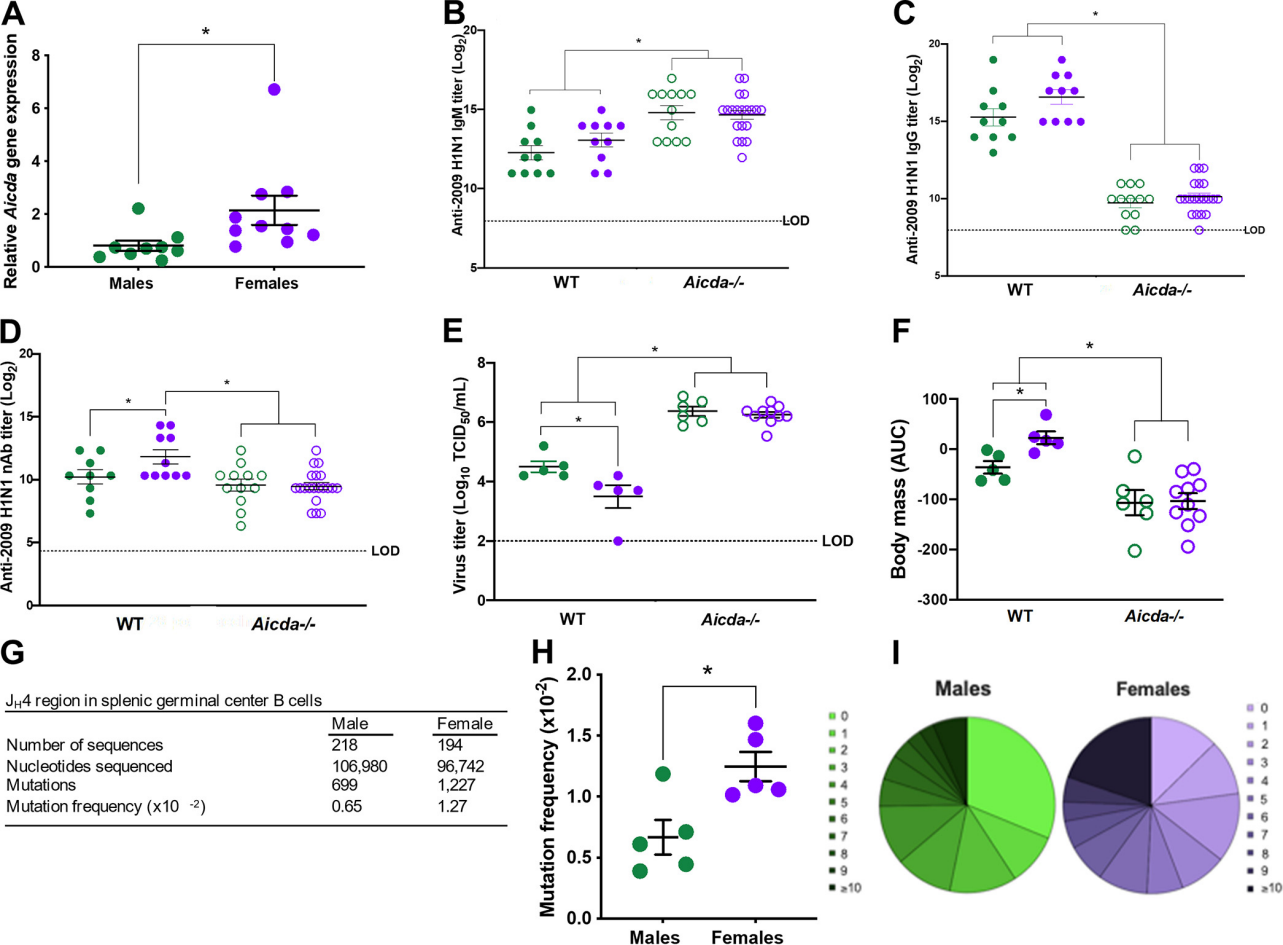
Greater dependence on and frequencies of somatic hypermutations in germinal center B cells mediate greater influenza vaccine-induced immunity and protection in female than male mice. Male and female C57BL/6CR were vaccinated and boosted with mouse-adapted A/California/4/2009 (maA/Cal/09) H1N1 vaccine virus. (A) Splenic B cells from male and female mice were isolated at 35 days postvaccination to measure activation-induced cytidine deaminase (*Aicda)* gene expression (*n* = 9 to 10/sex). (B to D) Serum from vaccinated and boosted wild-type (WT) and *AID^−/−^* male and female mice (*n* = 10 to 20/sex) was used to measure anti-maA/Cal/09 IgM (B), IgG (C), and neutralizing antibody (D) titers. (E) WT and *Aicda^−/−^* animals were challenged with 10^5^ TCID_50_ of the 2M mutant virus, and males and females were euthanized (*n* = 5 to 10/sex) at 3 days postchallenge to measure replicating virus titers in the lungs. (F) Vaccinated male and female mice (*n* = 5 to 10) were challenged with 10^5^ TCID_50_ of the 2M virus and were monitored for body mass loss over 14 days, with data converted into AUC values, with a lower AUC corresponding to greater mass loss. (G) Pooled antibody somatic hypermutation in germinal center B cells after vaccination in the J_H_4 intronic region was analyzed (*n* = 5/sex). (H and I) The mutation frequency in the J_H_4 intronic region of antibodies from splenic germinal center B cells collected 35 days after vaccination (H) and the percentages of total sequences containing increasing numbers of mutations per sequence are shown by the gradation of darker color in the pie chart (*n* = 5/sex) (I). Bars represent averages and SEM; LOD is the limit of detection for the assay. *, *P < *0.05.

Because class-switched IgG antibody titers and frequencies of GC B cells were greater among vaccinated females than males, we determined the mechanism of antibody maturation in females compared with males after vaccination. Expression of *Aicda* mRNA (i.e., the gene that encodes AID, which is a key enzyme in CSR and SHM antibody diversity reactions) was measured in B220^+^ B cells and was greater in B cells isolated from spleens of vaccinated females than males (Fig. 5A). To test the hypothesis that sex differences in vaccine-induced immunity and protection were dependent on elevated *Aicda* expression, WT and *Aicda^−/−^* male and female mice were vaccinated, and without the ability to undergo CSR, sex differences in antibody responses were eliminated in *Aicda^−/−^* mice, with both sexes producing greater titers of IgM (Fig. 5B) and lower titers of total IgG (Fig. 5C) than WT animals. Neutralizing capability toward the vaccine virus was significantly reduced in *Aicda^−/−^* versus WT females, but not for males (Fig. 5D). Finally, in the absence of antibody maturation, vaccinated *Aicda^−/−^* mice were more susceptible to lethal 2M challenge than WT mice. In the absence of AID, sex differences in vaccine-induced protection against pulmonary virus replication and morbidity (i.e., body mass loss) were eliminated (Fig. 5E and F).

During SHM, point mutations accumulate in the V gene sequences of the B cell receptor, a process that is regulated by AID and underlies antibody diversity (27). Because the elevated *Aicda* expression in total B cells could be caused by either greater frequencies of GC B cells or greater activity of AID in B cells from female mice, we examined if females had greater vaccine-induced SHM. To test this hypothesis, the J_H_4 intronic region of recombined V genes from GC B cells was sequenced to compare mutation frequencies. The overall number of mutations and corresponding mutational frequency were significantly greater in sequences from vaccinated females than males (Fig. 5G and H). Within the 218 regions sequenced from males and 194 regions sequenced from females, female mice had more mutations per sequence, with the majority displaying 10 or more mutations, whereas a greater percentage of male sequences were unmutated (Fig. 5I). These data suggest that highly mutated, class-switched antibodies afford females an advantage in terms of vaccine-induced protection against infection and disease after challenge with mutant maA/Cal/09 viruses.

## DISCUSSION

Females develop greater antibody responses to influenza vaccines than males (8, 15, 16, 18–20, 28, 29); there are, however, limits to female-biased protection following vaccination. The selected influenza vaccine strains have significant antigenic differences compared to circulating influenza viruses, which may be due to egg adaptation or other variables (4). In vaccine mismatch years, such as the 2017 to 2018 season in North America, the female-biased protection is lost (8). We characterized the limits to female-biased antibody responses and protection after vaccination, and we found that antibodies from vaccinated female mice were better able to recognize diverse HA epitopes and protect against mutant H1N1 viruses than antibodies from males. Unlike the well-accepted dogma that vaccine-induced nAb and IgG responses predict the degree of protection against influenza virus, our data show that this is not equally true for males and females. In females, heightened cross-recognition of mutant H1N1 viruses did not translate into heightened cross-neutralization to these viruses, as vaccinated male and female mice experienced equal neutralizing capabilities. In males, having equal nAb responses to mutant maA/Cal/09 viruses compared to females did not predict equal protection. Rather, the greater cross-reactivity of vaccine-induced IgG antibodies to diverse HA epitopes successfully correlated with greater protection from severe disease and pulmonary virus replication in females after live virus challenge with H1N1 mutants.

Cross-protection against mutant H1N1 viruses among females compared with males was dependent on B cells, as deletion of B cells either alone (*mu*MT mice) or in combination with T cells (*Rag1^−/−^* mice) eliminated female-biased protection following vaccination. Vaccination induced greater numbers and activity of GC B cells in females than males. The presence of functional B cells is necessary for female-biased protection. We also studied postvaccination GCs to understand how the GC reaction impacted female-biased protection. Vaccination induced greater numbers of GC B cells and greater expression of the SHM- and CSR-associated gene *Aicda* in B cells from vaccinated females than males. Within GC B cells, greater SHM frequencies were seen following vaccination of female compared with male mice. In the absence of antibody diversity (*Aicda^−/−^* mice), the female-biased immunity and protection were eliminated, illustrating that functional GC B cells and greater antibody diversity is necessary for the female bias in cross-protection against mutant H1N1 viruses. Increased expression of *Aicda* and increased frequency of SHM in GC B cells may in part be due to the numerous estrogen and progesterone response elements that have been mapped to the promoter region of the *Aicda* gene (30–33). Studies of antibody production in the context of autoimmune diseases have revealed that estrogen regulation of *Aicda* is critical for female-biased antibody production and disease progression (32). Supplementation of B cell cultures with exogenous estrogen increases IgG heavy chain transcription due to the presence of estrogen receptors in immunoglobulin regulatory elements and switch sites (34, 35). Whether estrogens or progesterone directly regulate SHM to improve vaccine-induced immunity is under investigation in our laboratory.

Limited studies have evaluated the impact of sex steroids on immune responses to vaccines. Estradiol, at physiological concentrations, can stimulate antibody production by B cells, including antibody responses to an IIV administered in mice (15, 36). In humans, reduced nAb responses to influenza vaccination are correlated with higher serum testosterone concentrations (16) and in mice, greater testosterone concentrations cause reduced antibody and CD8^+^ T cell activity following malaria vaccination (37). Elevated testosterone concentrations in males also are associated with greater lipid metabolism, suggesting that the immunosuppressive role of testosterone and the reduced antibody responses to vaccines in males may be mediated by the expression of genes involved in lipid metabolism that are associated with the suppression of inflammatory responses (16).

Drawbacks to this study include the inability to produce mutant viruses for each of the five major H1 antigenic sites, including point mutations in the Ca1 and Cb regions. Having more mutant viruses to diverse combinations of antigenic sites also would have allowed for estimation of sex differences in antibody and epitope usage or hierarchy, a phenomenon which is known to be different after influenza vaccination versus infection as well as different between mice and humans (23, 24, 38, 39). Whether males and females induce antibodies or nAbs to influenza viruses or influenza vaccine antigens in a differential hierarchical fashion is unknown. For this study, *Aicda* expression was measured in bulk B cells rather than GC B cells or other B cell types. Additional studies are required to interrogate which subtypes of B cells produce greater levels of AID and contribute to the greater SHM and CSR in females than in males. Also, GC reactions from the spleen rather than the draining LNs were evaluated due to the greater cell numbers and size of the spleen. Further studies should compare the GC responses in spleens to LNs in mice after vaccination, as organ-specific GC responses to influenza virus infection have been observed (40). Whether antibody affinity and avidity to the mutant H1N1 viruses display a sex-differential phenotype was not measured, and tracking the progression of both in germ line antibody compared to postvaccination antibody in males and females is required. Interrogation into the role of CD4^+^ T cells, particularly T follicular helper cells, in helping with induction of GC B cell numbers and activity is necessary. The formation of the differential GC response is dependent on cross talk between antigen, B cells, T cells, and antigen-presenting cells, which could be affected by the hormone milieu.

Understanding the relationship between biological sex, antigenic site immunodominance, and overall vaccine-induced protection against infection after influenza vaccination has implications for current IIV platforms and the development of universal influenza vaccines. Sex differences are eliminated in response to some of the universal influenza vaccine platforms. For example, vaccination with chimeric HAs results in equivalent avidity, antibody-dependent cellular cytotoxicity, total IgG, and IgG2c titers against HA stalk in response to the vaccine virus and other group 1 viruses among young adult male and female mice (41). Other universal influenza vaccine platforms have utilized mosaic H3 and influenza B virus HA approaches, in which entire antigenic sites on the head of HA are replaced with exotic HA sequences to increase antibody recognition to the more-conserved immuno-subdominant, yet more broadly protective, epitopes of HA after sequential immunization (42, 43). Unfortunately, only female mice were used in these studies. We predict that influenza vaccine strategies that induce both B and T cell help during GC formation may result in similar protection in males and females (44, 45). There also may be a sex-differential dependence on Fc-mediated effector mechanisms rather than nAbs in generating vaccine-induced responses. This could explain how vaccinated females experienced greater levels of cross-protection than males, despite having similar nAb titers. Antibodies can signal via their Fc domain, rather than Fab, to induce nonneutralizing antibody responses, which also play important roles in viral clearance (46). Vaccinated mice, for example, have been shown to be protected from infection via Fc-mediated antibodies to the matrix protein 2 (M2) (47). Continued exploration of how Fc-mediated antibody responses contribute to sex differences in vaccine-induced protection from influenza virus infection is necessary.

One of the most well-conserved immunological differences between the sexes is in antibody responses to foreign antigens (48). Beyond influenza vaccines, adult females have greater antibody responses to hepatitis B, yellow fever, rabies, herpes, and smallpox vaccines (48). Sex differences in antibody responses have evolved in diverse species, and we speculate that when offspring are young (i.e., during the neonatal period), protection against infection is primarily mediated by passive maternal immunity. In humans, a majority of maternal antibodies are transferred into fetal circulation, prior to birth, through the placenta (49). In some mammals, transfer of maternal antibodies can also occur through the colostrum immediately following birth and in milk for a longer duration after birth (49). Regardless of species, elevated production of antibodies caused by either vaccination or infection of females enhances the transfer of antibodies to the fetus or neonate to protect them during a critical period of infectious disease susceptibility. Natural selection may favor increased antibody production and the mechanisms therein, in females compared with males of reproductive ages because transfer of maternal antibodies from mother to young increases reproductive success by minimizing the detrimental effects of infection on offspring survival. While this might leave females more susceptible to antibody-mediated diseases, such as autoimmune diseases, these diseases typically cause morbidity after reproductive years and, therefore, would not be selected against. The next frontier will involve harnessing these observations and data to improve vaccines and equitable protection in both males and females.

## MATERIALS AND METHODS

### Mice.

Male and female C57BL/6CR mice (7 to 8 weeks of age) were purchased from Charles River Laboratories (Frederick, MD). Male and female wild-type (WT), *mu*MT, and *Rag1* knockout (*Rag1^−/−^*) mice on a C57BL/6J background (7 to 8 weeks of age) were purchased from the Jackson Laboratories (Bar Harbor, ME). The *Aicda* (activation-induced cytidine deaminase) knockout mice on a C57BL/6J background were maintained at the National Institute on Aging. Mice were housed at a maximum of 5 mice per cage under standard biosafety level 2 housing conditions with food and water available *ad libitum*. All animal procedures were approved by the Johns Hopkins University (ACUC protocol MO18H250).

### Recombinant virus generation.

The maA/Cal/09 challenge viruses contain mutations in the major antigenic regions of the HA head in the H0 following positions: one mutation (1M) at K180Q; two mutations (2M) at K180Q and G157E; three mutations (3M) at K180Q, G157E, and N211D; and a substitution (Sub) at K180Q with the entire Ca2 antigenic region substituted with a nonhuman H5 sequence (see Table S1 in the supplemental material). Recombinant viruses were generated using the IAV 12-plasmid reverse genetics system (50, 51). Recombinant maA/Cal/09, with mutations at HA sites D144E, S200P, and A212E (H0 numbering) to make the virus mouse-adapted, and 1M viruses were generated previously (19). HA escape mutations G157E on the Ca2 antigenic site, the N211D on the Sb antigenic site, and the Ca2 sequence substitution with H5 A/Vietnam/1203/2004 (23, 24) were introduced in pHH21-HA-maA/California/4/2009-K180Q to generate pHH21-HA, to make recombinant 2M, 3M, or Sub viruses by either site-directed mutagenesis (QuikChange Lightning site-directed mutagenesis kit, Agilent Technologies) or DNA synthesis (GenScript) (see Table S1). Eight maA/Cal/09 viral genome pHH21 plasmids with four helper plasmids expressing A/Udorn/72 (H3N2) PA, PB1, PB2, and NP proteins (51) were transfected into 50% confluent HEK293T cells in 6-well plates with TransIT-LT1 transfection reagent (Mirus Bio) according to the manufacturer’s protocol. After 24 h at 37°C with 5% CO_2_, 4 μL of 5 mg/mL of *N*-acetyl trypsin was added in each 6-well plate for 4 h at 37°C, 5% CO_2_. MDCKs (~5 × 10^5^ cells/well) with 20 μL/well of 30% bovine serum albumin (BSA) were then added and kept at 37°C, 5% CO_2_. Supernatants from the culture (1-mL aliquots) were collected and replaced with fresh infectious medium with 5 μg/mL of *N*-acetyl trypsin daily for 7 days and then titrated based on the TCID_50_. Virus positive collection at the earliest time point was purified using plaque assays on MDCK cells in 6-well plates. Plaque-purified viruses were expanded to seed stocks. The HA sequences of the seed stocks were confirmed using reverse transcription-PCR (RT-PCR) of viral RNA followed by Sanger sequencing.

### mLD_50_ assays.

Groups of immunologically naive mice were anesthetized with a ketamine-xylazine cocktail (80 mg/kg and 5 mg/kg) and inoculated intranasally with either 10^1^, 10^2^, 10^3^, 10^4^, 10^5^, or 10^6^ TCID_50_ of the maA/Cal/09, 1M, 2M, 3M, and Sub viruses. Survival was monitored for 21 days, and the mLD_50_ for each sex for each virus was calculated via the Reed-Muench method (20, 52). If mice lost 30% or more of their mass, they were humanely euthanized.

### Mouse vaccination, challenge, and morbidity.

Mice received an intramuscular injection of 20 μg of maA/Cal/09 inactivated vaccine in 40 μL of 1× phosphate-buffered saline in the right thigh muscle for the prime (day 0) and boost (day 21) vaccinations. For live virus challenge, mice were anesthetized with a ketamine-xylazine cocktail and inoculated intranasally 6 weeks after vaccine prime (42 days postvaccination) with 10^5^ TCID_50_ of the 1M, 2M, or Sub virus suspended in 30 μL of Dulbecco modified Eagle medium. Changes in mouse body mass were measured daily, with percent body mass loss values over time translated into AUC to capture the changes over time into individual data points.

### Virus quantification in mouse lungs.

Lungs were collected from mice at several time points post-live viral challenge. Lungs were homogenized and serially diluted in infectious media (see Text S1) before being plated on a monolayer of 100% confluent MDCK cells in replicates of 6. Cells were left to incubate for 6 days at 32°C and 5% CO_2_. Cells on plates were fixed for 1 h in 4% formaldehyde and then stained with naphthol blue black for at least 4 h. Plates were scored for cytopathic effect, and the Reed-Muench method (52) was used to calculate the TCID_50_ values for the lungs of each animal.

### Statistical analyses.

Graphs were created and statistics were performed in GraphPad Prism 9. Because comparison groups had equal variance and normal distributions, parametric statistics were used. A *t* test (to compare between two groups), one-way analysis of variance (ANOVA; to compare among three or more groups with a single independent variable), and two-way ANOVA (to compare between groups when two variables) were used to analyze plaque sizes, IgM, total IgG, and IgG2c titers, nAb titers, virus titers, changes in body mass, cell numbers, GC sizes, mutation frequencies, and gene expression data. For one- and two-way ANOVAs, analyses were followed by Tukey’s multiple-comparison tests. Differences were considered significant if *P *was ≤0.05.

### Data availability.

All data will be made available upon request. Somatic hypermuation sequencing data are publicly available at Mendeley Data doi: 10.17632/26hj2zrdxf.1.
