# Supplementary material for: Greater Breadth of Vaccine-Induced Immunity in Females than Males Is Mediated by Increased Antibody Diversity in Germinal Center B Cells
Source: mBio. 2022 Jul 20;13(4):e01839-22. doi: 10.1128/mbio.01839-22 (PMC9426573; doi:10.1128/mbio.01839-22)
Supplement: TABLE S1 [file mbio.01839-22-s0004.docx]

**
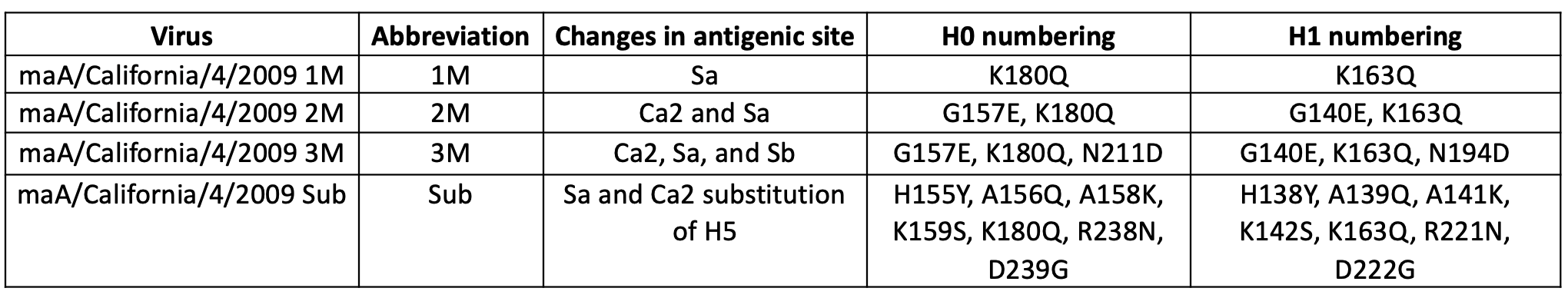
**

**Table S1 Legend: Amino acid mutations on mouse-adapted A/California/4/2009 H1N1 virus hemagglutinin protein used in this study.** The parental mouse-adapted A/California/4/2009 (maA/Cal/09) H1N1 influenza virus was used to create four mutant viruses with either one, two, or three single point mutations in the hemagglutinin (HA) head (1M, 2M, 3M respectively), or a virus with an entire antigenic region substituted and replaced with a non-human H5 sequence (Sub). The antigenic sites affected by the mutations as well as the amino acids and numbering (for both H0 and H1) are listed for each mutant virus.
