# Supplementary material for: Greater Breadth of Vaccine-Induced Immunity in Females than Males Is Mediated by Increased Antibody Diversity in Germinal Center B Cells
Source: mBio. 2022 Jul 20;13(4):e01839-22. doi: 10.1128/mbio.01839-22 (PMC9426573; doi:10.1128/mbio.01839-22)
Supplement: TEXT S1 [file mbio.01839-22-s0001.docx]

**Supplemental Materials and Methods**

**Plaque Assays**

MDCK cells were grown to 100% confluency in complete medium (CM; DMEM, fetal bovine serum (FBS), L-glutamine, and Pen-strep) in 6-well plates (1-3). The CM was removed, cells were washed with phosphate buffered saline (PBS)+ (1xPBS with 2mm calcium and magnesium) twice, and 400µL of virus inoculum was added to cells. Plates of cells were incubated at 32$℃$ for 1 h with rocking. The virus inoculum was removed after 1 hour of incubation and phenol-red free DMEM supplemented with 3% BSA, 100U/ml pen/strep, 2mM Glutamax (Gibco), 5µg/ml *N*-acetyl trypsin, and 1% agarose was added. Plates of cells were then incubated for 3-5 days at 32$℃$ and fixed with 4% formaldehyde. The topmost agarose was removed, and cells were stained with naphthol-blue black (Sigma Aldrich). Plaque size was analyzed in Image J (4). For the 1M, 2M, 3M, and Sub recombinant virus production, virus plaques were picked, placed in media, and stored at -80$℃$ for virus seed stocks. To calculate plaque forming units (PFU/mL) for each virus, the number of plaques were counted and divided by the given dilution used to seed virus.

**Low-MOI Virus Growth Curves**

Low-MOI (multiplicity of infection) growth curves were performed (2, 3), at a MOI of 0.001 on MDCK cells, which were infected in infectious media (IM; DMEM, 10% BSA, 5% L-glutamine, 5% pen-strep, and 5µg/mL *N*-acetyl trypsin) at 32$℃$ for 1 h. After infection, the virus inoculum was collected, cells were washed three times with 1xPBS+, new IM was added, and cells were placed at 32$℃$. At 1-, 12-, 24-, 36-, 48-, 72-, and 96-hours post-inoculation, IM was removed from the MDCK cells and stored for TCID_50_ infectious virus quantification. After each time point, fresh IM was added. For virus titers measured over time, the titer values at each time point were used to create a curve which was used to calculate area under the curve (AUC) as a representation of overall growth over time.

**Generation of Vaccine and ELISA Proteins**

The maA/Cal/09 H1N1 virus (generated from available sequences (5)) was maintained and grown in IM on MDCK cells. To generate vaccine, maA/Cal/09 virus was grown at an MOI of 0.01 in MDCK cells for three days, upon which virus supernatant was collected, centrifuged, and inactivated with the addition of 0.05% β-propiolactone for 24 h followed by a 2 h 37$℃$ incubation. Heat and chemically inactivated virus was purified by ultracentrifugation in a Beckman SW28TU rotor at 25,000rpm for 1 hour at 4$℃$ with a 20% sucrose gradient and the virus pellet was resuspended in 1xPBS+. To confirm virus inactivation, TCID_50_ was performed. ELISA protein for maA/Cal/09, 1M, 2M, 3M, and Sub was grown similarly with no inactivation steps. A bicinchoninic acid assay (BCA, Pierce) was used to estimate viral protein concentration.

**Enzyme-Linked Immunosorbent Assays (ELISAs)**

Anti-maA/Cal/09, 1M, 2M, 3M, or Sub H1N1 total IgG and IgG2c antibody titers were measured in plasma (6). ELISA plates (Microlon 96 well high binding plates; Greiner Bio-One) were coated with 100ng of purified virus for 24 h at 4$℃$ in carbonate bicarbonate buffer (pH 9.6). Secondary antibodies for either anti-mouse horseradish peroxidase (HRP)-conjugated IgG (1:250, Invitrogen), IgM (1:3,000, Invitrogen), or IgG2c (1:20,000, Thermo Fisher Scientific) were added for 1 h at 37$℃$. After development, the absorbance was read at 450nm, and titers were calculated as the highest serum dilution with an average OD value above 3 times the average OD of the negative controls.

**Microneutralization Assays**

The anti-maA/Cal/09, 1M, 2M, 3M, and Sub H1N1 neutralizing antibody (nAb) response in plasma was determined as described previously (6). Plasma was heat inactivated for 35 min at 57$℃$ before use. Plasma was diluted two-fold in IM and combined with 100 TCID_50_ of the maA/Cal/09, 1M, 2M, 3M, or Sub H1N1 virus for 1 hour at room temperature. The combined plasma and virus were added in duplicate to confluent MDCK cells for 24 h at 32$℃$. Plates were washed, new IM was added, and plates were incubated at 32$℃$ for 6 days, then fixed with 4% formaldehyde for 1 h, and stained with naphthol blue black for 4 h. The nAb titer was calculated as the highest serum dilution that prevented cell death in half of the wells.

**Phage ImmunoPrecipitation Sequencing (PhIP-Seq) and Analysis**

PhIP-Seq, using mouse serum, was performed as described (7). Briefly, 20μL of 1:100 diluted plasma in 1xPBS from individual mice were mixed with a 56 amino acid peptide library that tiles across the proteins of all human viruses (VirScan) (8), then immunoprecipitated using protein A and protein G coated magnetic beads. Eight “mock” immunoprecipitations (IPs, no serum input) was run on the same plate. Beads were washed and resuspended in PCR master mix, 20 cycles of PCR were performed, followed by 20 more cycles of PCR with sequencing adapter and sample barcode containing primers. Amplicons were pooled and sequenced on an Illumina instrument using a 1x50 cycle protocol.

Sequencing reads were mapped to the VirScan library using perfect matching. By comparing against the set of mock IPs, a fold change and differential abundance statistic were calculated for each peptide in each sample using the edgeR Bioconductor package (9). Peptides were considered “hits” for a sample if the following conditions were met: count ≥ 15, p-value ≤ 0.001, and fold-change ≥ 5. Hits fold-change (HFC) values report the fold-changes of hits and is set to 1 for non-hits. Only the HA peptides from IAVs were considered in this study.

**Flow Cytometry and FACS**

The numbers of GC B cells in the spleen were estimated using flow cytometry (6). At 35 days post vaccination, a single splenocyte suspension was made in FACS buffer (1xPBS, 1% heat inactivated FBS, 25mM HEPES, and 1mM EDTA) and filtered. Red blood cell lysis was performed using ACK lysis buffer. The Fc receptors in each sample were blocked using anti-CD16/32 (BD Biosciences) The total number of live cells were counted using a Cellometer Auto 2000 (Nexcelom Bioscience) with an AOPI ViaStain solution (Nexcelom Bioscience). An antibody cocktail for GC B cells (B220+CD38-GL7+) was added and incubated for 20 min at 4$℃$ in the dark. Antibodies included rat anti-mouse PE-Cy7-conjugated CD45R/B220+ (BD Biosciences), rat anti-mouse FITC-conjugated GL7 (BD Biosciences), and rat anti-mouse BV421 CD38 (BD Biosciences). Cell numbers were acquired using either FACSAria (BD Biosciences) or a MoFlo XPD High Speed Cell Sorter (Beckman Coulter) and analyzed using FlowJo v.10 (Tree Star, Inc.). GC B cells were sorted using a BD FACS Aria Fusion (BD Biosciences).

**Immunofluorescence and Confocal Microscopy**

Spleens were mixed with 10% and then 30% sucrose to remove water and embedded in OTC and frozen at -80$℃$. Serial 10µm thick sections were taken from the frozen tissue block using the cryotome and slices were fixed onto slides with cold acetone for 10 min. Slides were washed with 1xPBS and incubated in 0.3% H_2_O_2_ solution for 10 min. After blocking with 0.1% titronX-100 in 5% BSA for 1h at RT, a PNA-biotinylated antibody (Vector Bio) was applied at 4$℃$ overnight. Slides were washed and incubated with Alexa Fluor 488 streptavidin (Biolegend) as a secondary antibody and Alexa Fluor 647 anti-mouse IgD (Biolegend). Sections were washed one final time and mounted with a DAPI solution and covered with glass and stored at -80$℃$.

Confocal fluorescent images were acquired on a Zeiss Axio Observer.Z1 (Zeiss) fluorescent microscope with Colibri.2 LED light source and an ORCA-R2 digital CCD camera (Hamamatsu) using ZEN imaging software. Tiling and stitching function was used to generate high-resolution images that covered entire spleen sections in a continuous field using a 5X objective. For each spleen, 3 spleen sections were imaged. Individual GCs were imaged at 10X magnification, and the relative area of each GC was calculated by measuring the quotient of the GC area (PNA+, stained green in images) over the entire B cell area (IgD+, stained magenta in images) using Fiji software (ImageJ).

**B cell isolation for real-time RT-PCR**

All CD19+ B cells were isolated (STEMCELL Technologies EasySep Mouse B Cell Isolation kit, STEMCELL Technologies) and mRNA was extracted and purified (PureLink RNA Mini Kit, Invitrogen). This mRNA was used to generate cDNA via RT-PCR. *Aicda* expression was measured by qPCR (Primers from Integrated DNA Technologies, Mm.PT.58.42247522). The relative gene expression was normalized to a *Gapdh* house-keeping gene and mock-vaccinated animals using the ΔΔCT method (6).

**Somatic Hypermutation Intron Sequencing and Analysis**

Splenic GC B cells (B220+CD38-GL7+) were sorted. Cells were lysed in digestion buffer (10 mM Tris, pH 8.0, 25 mM EDTA, 100 mM NaCl, 1% SDS, and 0.1 mg/ml proteinase K) at 55°C overnight. Genomic DNA was isolated by phenol/chloroform extraction and ethanol precipitation. The J_H_4 intronic region was amplified using a nested PCR protocol with Herculase II high-fidelity polymerase (Agilent). Primary PCR was performed for 25-cycles using V-region forward primer (5’-AGCCTGACATCTGAGGAC-3’) and intron reverse primer (5’-GAGCCTCACTCCCATTCCTCGG-3’), followed by a 35-cycle secondary PCR using V-region primer (5’-GCCTGACATCTGAGGACTCTGC-3’) and intron reverse primer (5’-TAGATGCCTTTCTCCCTTGACTCA-3’). The 492 bp of J_H_4 intronic DNA was sequenced and unique VDJ clones were analyzed for mutations.

**References**

1. Wohlgemuth N, Lane AP, Pekosz A. 2018. Influenza A Virus M2 Protein Apical Targeting Is Required for Efficient Virus Replication. J Virol 92.

2. Liu H, Grantham ML, Pekosz A. 2018. Mutations in the Influenza A Virus M1 Protein Enhance Virus Budding To Complement Lethal Mutations in the M2 Cytoplasmic Tail. J Virol 92.

3. Powell H, Liu H, Pekosz A. 2021. Changes in sialic acid binding associated with egg adaptation decrease live attenuated influenza virus replication in human nasal epithelial cell cultures. Vaccine 39:3225-3235.

4. NIH. 2020. ImageJ. <https://imagej.nih.gov/ij/>. Accessed

5. Ye J, Sorrell EM, Cai Y, Shao H, Xu K, Pena L, Hickman D, Song H, Angel M, Medina RA, Manicassamy B, Garcia-Sastre A, Perez DR. 2010. Variations in the hemagglutinin of the 2009 H1N1 pandemic virus: potential for strains with altered virulence phenotype? PLoS Pathog 6:e1001145.

6. Fink AL, Engle K, Ursin RL, Tang WY, Klein SL. 2018. Biological sex affects vaccine efficacy and protection against influenza in mice. Proc Natl Acad Sci U S A 115:12477-12482.

7. Mohan D, Wansley DL, Sie BM, Noon MS, Baer AN, Laserson U, Larman HB. 2018. PhIP-Seq characterization of serum antibodies using oligonucleotide-encoded peptidomes. Nat Protoc 13:1958-1978.

8. Xu GJ, Kula T, Xu Q, Li MZ, Vernon SD, Ndung'u T, Ruxrungtham K, Sanchez J, Brander C, Chung RT, O'Connor KC, Walker B, Larman HB, Elledge SJ. 2015. Viral immunology. Comprehensive serological profiling of human populations using a synthetic human virome. Science 348:aaa0698.

9. Robinson MD, McCarthy DJ, Smyth GK. 2010. edgeR: a Bioconductor package for differential expression analysis of digital gene expression data. Bioinformatics 26:139-40.
