# Supplementary material for: Greater Breadth of Vaccine-Induced Immunity in Females than Males Is Mediated by Increased Antibody Diversity in Germinal Center B Cells
Source: mBio. 2022 Jul 20;13(4):e01839-22. doi: 10.1128/mbio.01839-22 (PMC9426573; doi:10.1128/mbio.01839-22)
Supplement: FIG S2 [file mbio.01839-22-s0003.docx]

**
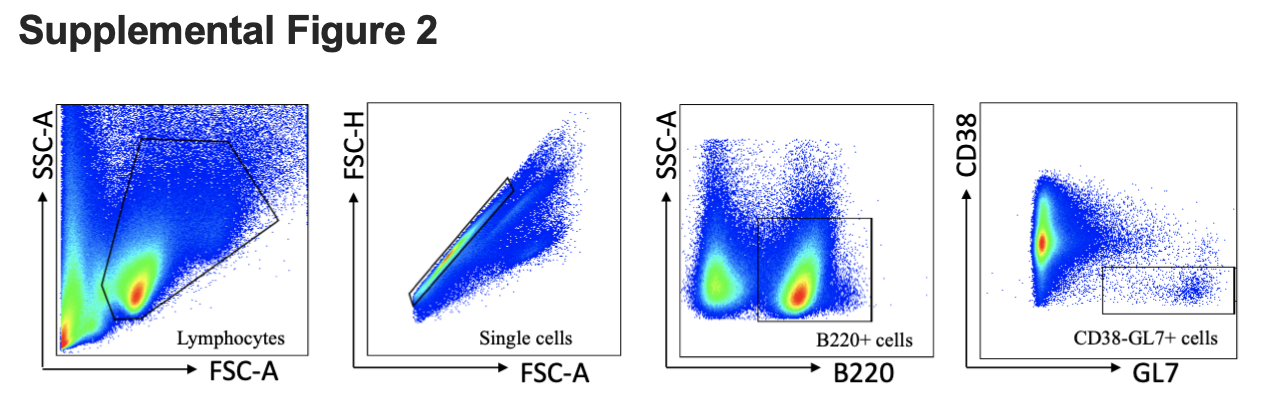
**

**Fig. S2 Legend: A representative flow cytometry gating strategy used to quantify germinal center B cells from mouse spleen.** Male and female wild type (WT) C57BL/6CR mice were vaccinated and boosted with mouse-adapted A/California/4/2009 (maA/Cal/09) H1N1 virus. At 35 days post vaccination, spleens were collected, and cells were prepared as described in Methods. Flow cytometry was performed to measure the total number and frequency of germinal center (GC) B cells and to isolate GC B cells for downstream somatic hypermutation analysis. Representative flow plots are shown, with gates set for (from left to right) total live lymphocytes, single cells, B220+ B cells, and subsequently GC B cells marked as CD38-GL7+.
